# Anti-malarial seroprevalence assessment during an elimination programme in Chabahar District, south-eastern Iran

**DOI:** 10.1186/s12936-016-1432-1

**Published:** 2016-07-22

**Authors:** Sedigheh Zakeri, Lotus Leonie van den Hoogen, Akram Abouie Mehrizi, Fatemh Karimi, Ahmad Raeisi, Chris Drakeley

**Affiliations:** Malaria and Vector Research Group (MVRG), Biotechnology Research Center (BCR), Pasteur Institute, P.O. Box: 1316943551, Tehran, Iran; Department of Immunology and Infection, London School of Hygiene & Tropical Medicine, London, UK; National Programme Manager for Malaria Control, Ministry of Health and Medical Education, Tehran, Iran; School of Public Health, Tehran University of Medical Sciences, Tehran, Iran

## Abstract

**Background:**

Iran has achieved a substantial decline in malaria incidence over the past decades. A common feature of malaria-endemic settings is the requirement for more sensitive techniques to describe levels of low transmission. In this study, serological and parasitological methods were used to measure transmission levels of *Plasmodium falciparum* and *Plasmodium vivax* during an elimination programme (2012) in Chabahar District, Sistan and Baluchistan Province, south-eastern Iran.

**Methods:**

Participants were randomly selected from 64 different geographical clusters in Chabahar city and surrounding villages. Antibody responses to *P. falciparum* and *P. vivax* blood-stage antigens were assessed by ELISA, while microscopy and molecular testing were used to determine parasite carriage by species. Age-adjusted antibody responses were analysed using a reversible catalytic model to calculate seroconversion rates (SCR).

**Results:**

There was no evidence of recent transmission in the study areas, indicated by an absence of parasite infections in all ages and low or absent serological responses to either species in young children. The best model for age *P. falciparum* seroconversion was one with a change in exposure 21 years before sampling was done in Chabahar city (*P* = 0.018) and 4 years in the villages (*P* = 0.039). There was a higher level of recent *P. vivax* transmission compared to *P. falciparum*, based on the SCRs, in both the city and village settings.

**Conclusion:**

Serological analysis identified a decline in *P. falciparum* transmission in the urban areas of Chabahar, consistent with a previously described decrease in malaria in the early 1990s, demonstrating the utility of this approach to reconstruct exposure history. At present, it remains unclear whether the *P. vivax* antibody responses reflect active transmission due to new infections or relapse infections. The absence of parasitological and serological evidence of recent malaria transmission in Chabahar District is viable evidence for certification of elimination.

**Electronic supplementary material:**

The online version of this article (doi:10.1186/s12936-016-1432-1) contains supplementary material, which is available to authorized users.

## Background

Following global decline in malaria over past decades [1], the importance of accurately describing transmission in (pre-) elimination areas is widely documented [2]. Use of data collected at health facilities might not be robust in some areas as data are highly dependent on health-seeking behaviour and the effectiveness of the health system [3]. Evaluating transmission intensity by determining exposure to malaria-infected mosquitoes is challenging when mosquito numbers are low [4]. Serological evidence of exposure to malaria, by the presence of anti-malarial antibodies, offers a measure of past infection and can determine temporal [5] as well as spatial trends in transmission [6]. In low-endemic settings, long-lasting antibody responses may be easier to detect than parasite carriage in the human population or infected mosquitoes. Moreover, the absence of anti-malarial antibodies in certain age groups has been used as evidence of the cessation of transmission in Greece and Mauritius [7, 8]. The presence of serological evidence of exposure to malaria alongside molecular testing to detect parasites are currently considered to be most appropriate metrics in areas of low transmission and under elimination [3, 9].

In Iran, a national strategic plan for the elimination of malaria was approved by High Council for Health and Food Security in 2010 to stop local transmission by 2025. In this regard, Iran achieved a substantial decline in malaria incidence according to WHO Malaria Reports [1, 10]. The burden of malaria decreased gradually from nearly 97,000 cases in 1991 to 16,000 in 2007 [Center for Diseases Management and Control (CDMC), Tehran, Iran, unpublished data]. A further reduction was reported in 2014 with 1251 being the total number of cases (75 % decrease since 1991). Iran is considered to be in the elimination phase for malaria since 2009 [11]. Areas of malaria transmission are found in the southern part of the country, which accounts for more than 90 % of the total number of cases [12]. The majority of cases, 70 % in 2014 (CDMC, Tehran, Iran, unpublished data), is due to imported cases from neighbouring Pakistan and Afghanistan [13].

Investigating the application of different metrics in order to demonstrate the absence of transmission and simultaneously show the likelihood of success and outcomes of malaria interventions during pre-elimination and elimination programmes is essential in Iran. Previous serological studies in Bashagard and Ghale-Ganj Districts, southern Iran, found seroprevalence to *Plasmodium vivax* and *Plasmodium falciparum* to be 1 % or less [12, 14]. No parasite carrier was detected by microscopy and/or molecular testing. Although seroprevalence was low in these districts, higher transmission patterns in neighbouring Sistan and Baluchistan Province are to be expected, based on previous incidence data [1]. This study was designed to determine serological and parasitological transmission levels of *P. vivax* and *P. falciparum* malaria in Chabahar District, Sistan and Baluchistan Province in the malaria elimination phase in southeastern Iran.

## Methods

### Study areas and sample collection

**Fig. 1:**
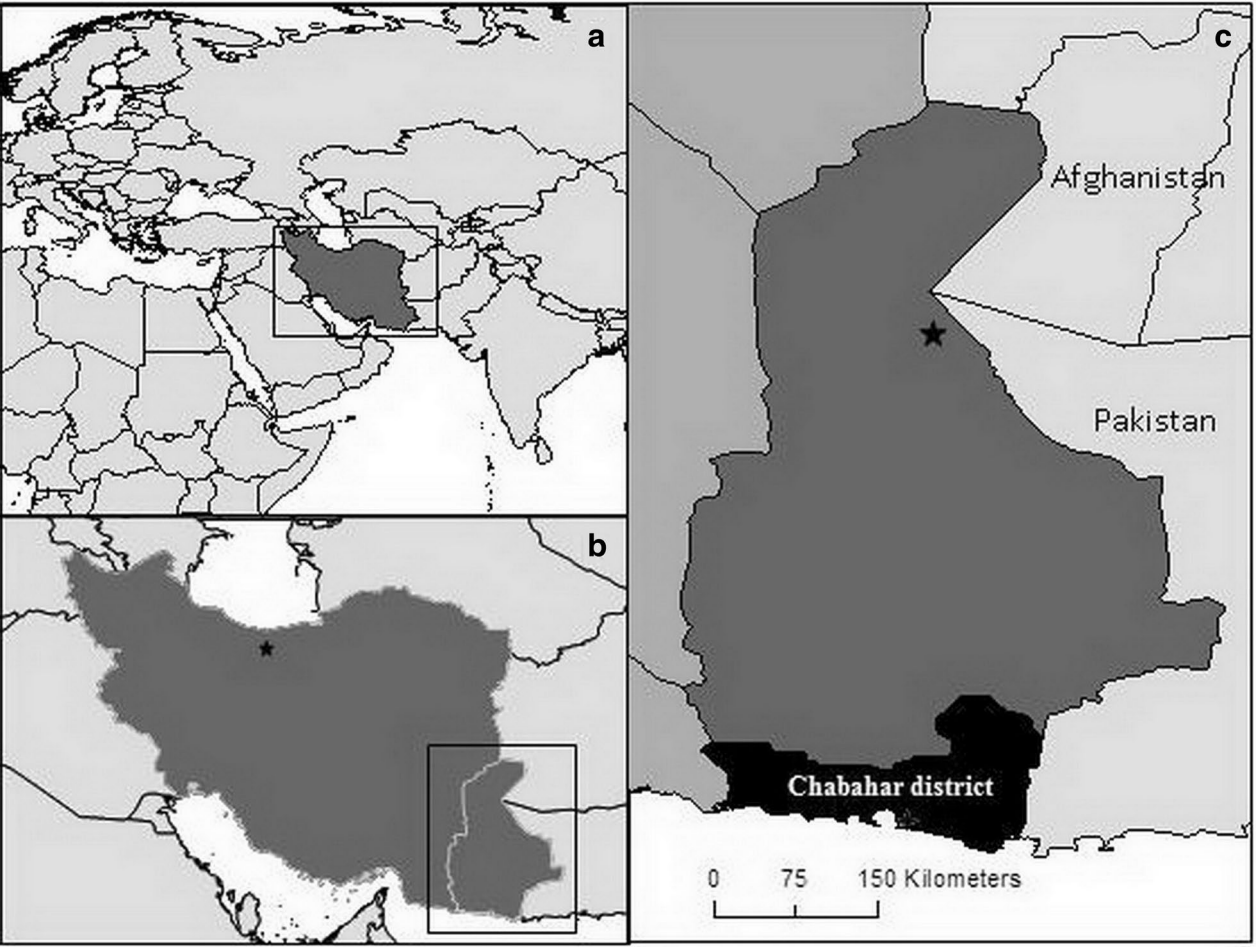
Map of **a** Iran and surrounding countries, **b** Iran with capital Tehran (*star*) and **c** Sistan and Baluchistan Province with capitals Zahedan (*star*) and Chabahar District in *black*. In **c** Sistan and Baluchistan Province are indicated in *dark grey*, other provinces in Iran in *mid grey* and neighbouring countries Afghanistan and Pakistan in *light grey*

This cross-sectional study was performed in Chabahar District, Sistan and Baluchistan Province in the southeastern part of Iran in March 2012 (Fig. 1). Based on the 2014 available census the total population of Chabahar city was 120,000 with a total approximate population of 200,000 in the county of Chabahar. In these areas, both *P. falciparum* and *P. vivax* exist; however, *P. vivax* is the dominant species with more than 88 % malaria infection cases (CDMC, Iran, unpublished data, 2014). The malaria cases reported in Iran was 3239 in 2011, 1629 cases in 2012 and 1387 in 2013 [15, CDMC, Tehran, Iran, unpublished data]. In this area, most of the patients are adults and they may experience several infections by *P. falciparum* and *P. vivax*; however, there is no record of severe cases or deaths due to malaria (CDMC, Tehran, Iran, unpublished data). In this study, participants were selected from 64 different geographical clusters, (previously described endemic areas based on malaria incidence data [1]) in Chabahar city and surrounding villages. In Chabahar city participants were selected from 32 clusters targeting hot spots areas based on reported malaria case data. In rural areas 32 villages were selected across four groups: high (n = 10), moderate (n = 8), low (n = 6) and absent (n = 8) malaria cases during the year 2011. In each cluster, 24 subjects were selected evenly from both sexes and from four age groups (<5, 6–15, 16–45, >45 years old), using systematic random sampling methods.

Demographic (e.g., age, gender, travel history, use of long-lasting insecticide nets (LLIN) and household information (e.g., wall and ceiling structures) were obtained using a structured questionnaire, and measured axillary temperatures over 37.5 °C were recorded. Information on history of malaria infection was obtained through the questionnaire and confirmed by local healthcare documents. During house visits, entomologists examined any water source around the house that could be a potential larval breeding site for the presence of egg, larvae or pupae of *Anopheles* mosquito species. Houses located within 100 m of a larval breeding site were recorded. Finger-prick blood samples were obtained from each participant for microscopy smears as well as filter-paper blood spots, and the latter were stored at −20 °C with desiccant until processing. Blood slides were taken back to health centres and read by a malaria microscopy expert and in case individuals tested positive, they were treated through direct observed treatment by health workers at home.

### Assessment of parasite carriage by microscopy and nested-polymerase chain reaction

Thick and thin blood smears were considered negative if no parasites were observed after examining 200 fields of Giemsa-stained thick blood smears or by counting 1000 red blood cells in thin blood smears at 1000× magnification by an expert microscopist. To increase the sensitivity of parasite detection, all obtained results by microscopy were confirmed by nested polymerase chain reaction (nPCR). Briefly, two 3-mm spots were cut from filter papers using a puncher. The QIAamp^®^ DNA Mini Kit (QIAGEN Inc, Valencia, CA, USA) was used to extract DNA according to the manufacturer’s instructions. *P. vivax* and *P. falciparum* was detected by nested-PCR amplification of the small sub-unit ribosomal ribonucleic acid (18ssrRNA) genes. The nest-1 PCR was performed similarly for both *Plasmodium* species using primers described previously [16]. Amplification was carried out in a final volume of 25 µL including 1 µL nest-1 PCR product as a template. The cycling condition for nest-2 PCR of *P. vivax* and *P. falciparum* corresponding genes was as follows: 95 °C for 5 min, 25 cycles of 94 °C for 1 min, 58 °C for 1 min, 72 °C for 1 min followed by 72 °C for 15 min. Positive control samples, as well as three negative controls, were included in each set of amplification reactions. All products were visualized in 2 % agarose gels containing ethidium bromide under ultraviolet transillumination (UVItec, Cambridge, UK).

### Assessment of anti-malarial antibody responses

Blood spots were reconstituted as previously described and the solution was equivalent to a 1:100 dilution of whole blood or a 1:200 dilution of serum [17]. Antibody responses against apical membrane antigen-1 or the 19 kDa fragment of merozoite surface protein-1 for *P. vivax* (PvAMA-1 and PvMSP-1_19_, respectively) and *P. falciparum* (PfAMA-1 and PfMSP-1_19_) were determined by ELISA as previously described [17]. Briefly, sera samples were added in duplicate at a final concentration of 1:1000 for MSP-1_19_ and 1:2000 for AMA-1. In addition, four blank wells and a five-fold dilution series (starting at 1:100 for AMA-1 and 1:50 for MSP-1_19_) of an African hyper-immune plasma pool (n = 30) were added per plate. Optical density (OD) values were measured at 490 nm with a microplate reader.

### Statistical analyses

Duplicate ODs per individual were averaged, adjusted for background reactivity and normalized against the positive control curve as previously described to adjust for plate variation [17]. All further statistical analyses were done in STATA version 14. Infants under 1 year of age were excluded to remove any influence of maternally derived antibodies [18]. Thresholds to define seropositivity for the separate antigens were calculated using a finite mixture model [19]. Individuals were defined as seropositive when their adjusted OD value would be greater than the mean of the lower Gaussian distribution plus three times the standard deviation.

Principal component analysis was used to combine the following variables reflecting socio-economic status (SES) as previously described by Vyas et al. [20]: house with cement walls, a plaster ceiling, connected to pipeline water, connected to electricity, ownership of a domestic animal, ownership of additional facilities (such as a TV/fan/car), and family size. Three levels of SES were created (low, middle, high) and this was done separately for those living in the city and those in the surrounding villages. Details on the distribution of these variables by SES status can be found in Additional files 1 and 2.

Differences in continuous descriptive metrics between Chabahar city and surrounding villages were tested with the Wilcoxon Mann–Whitney test, while differences in proportions were tested with Chi squared tests. Differences in proportions were adjusted for the clustered sampling using the svy command in STATA without weighting by cluster size. The reversible catalytic model was used to define the seroconversion rate (SCR) and to plot corresponding seroconversion curves while fitting age-adjusted seropositivity to *P. falciparum* or *P. vivax* using maximum likelihood [18]. The SCR is analogous to the force of infection and reflects the rate at which the population becomes seropositive. The presence of a change point in transmission was investigated during the same model, yet fitting two different SCRs in the same population. Profile likelihood plots were used to determine when the most likely time for change in transmission intensity occurred. The model with a change point was compared to the model without a change point using the likelihood ratio test and the model that fitted the data best was presented [19].

Logistic regression was used to identify the likelihood of seropositivity based on explanatory factors. Standard errors were adjusted to allow for the clustered sampling as described above. An adjusted model was also created for all explanatory factors. Using retrospective sample size calculation methods for serology, considering the approximate 750 samples per study setting, an SCR of 0.004 with a 95 % confidence interval can be estimated with a width of 0.002 to 0.004 (equating to an entomological inoculation rate of 0.00–0.02) [21].

## Results

### General characteristics of the study population and parasite detection

Serum was successfully eluted and serologically tested for 1479 participants and they were evenly distributed between the city (744, 50 %) and surrounding villages (735, ~50 %). Median age (16 years, ranging 2–70 and 15, ranging 2–78) were similar in the two studied settings (*P* = 0.357, Table 2). Risk factors for malaria were unevenly distributed (Table 1): LLIN use was significantly lower in the village compared to the city (24 vs 68 %; *P* < 0.001) and reported history of malaria was significantly higher (24 vs 10 %; *P* = 0.003). In the city, participants were more likely to live close to a mosquito-breeding site compared to villagers (89 vs 53 %; *P* = 0.001). All participants were parasite-negative both by microscopy and nPCR.

**Table 1:** General characteristics of the study population in Chabahar city and surrounding villages, south-eastern Iran

Demographic data	% (95% CI)		*P* value
	City (n = 744)	Village (n = 735)	
Median age (range)	16 (2–70)	15 (2–78)	0.357
Male	42 % (38–45)	51 % (44–57)	0.012
Fever (≥37.5 °C)	–	0 % (0–1)	0.159
LLIN	68 % (56–78)	24 % (13–40)	<0.001
History of malaria^a^	10 % (7–14)	24 % (18–31)	0.003
History of travel^b^	13 % (7–25)	5 % (3–8)	0.017
Breeding site^c^	89 % (76–95)	53 % (37–69)	0.001
High population density^d^	77 % (61–88)	49 % (34–64)	0.026
Visitor from endemic area^e^	12 % (5–23)	12 % (6–23)	0.906

All reported proportions are adjusted for clustering within enumeration areas

^a^Reported history of malaria over the past 10 years; confirmed by local health records

^b^Reported travel history 4 weeks prior to sampling

^c^Within a 100 m radius of the household

^d^More than ten houses within a 100 m radius of the household

^e^Within the last 2 weeks

### Trends in anti-malarial antibody responses by age

To define any serological evidence of exposure to *P. falciparum* or *P. vivax*, seropositivity to species-specific AMA-1 and MSP-1_19_ antigens was combined. Participants living in villages were more likely to show evidence of exposure to either species compared to city dwellers. In total, 15 % of villagers were seropositive for *P. falciparum* (109/735), while 13 % for *P. vivax* (95/735) and 4 % for both species (30/735). In the city, 11 % (78/744), 7 % (50/744) and 2 % (14/744) were seropositive, respectively (Table 2).

**Table 2:** Anti-malarial seropositivity and seroconversion rates for participants in Chabahar city and surrounding villages, south-eastern Iran

	Prevalence, % (n/N)					
	City			Village		
	*P. falciparum*	*P. vivax*	*P.f/P.v*	*P. falciparum*	*P. vivax*	*P.f/P.v*
1 to 5	2 (4/174)	4 (7/174)	1 (1/174)	0 (0/159)	5 (8/159)	0 (0/159)
6 to 15	3 (5/191)	7 (14/191)	1 (2/191)	8 (16/213)	9 (19/213)	1 (3/213)
>15	19 (69/368)	8 (29/368)	3 (11/368)	26 (92/357)	19 (67/357)	8 (27/357)
All ages^a^	11 (78/744)	7 (50/744)	2 (14/744)	15 (109/735)	13 (95/735)	4 (30/735)
SCR (λ)	λ_1_: 0.004 (0.002–0.007)	0.014 (0.006–0.032)		λ_1_: 0.000 (0.000–0.000)	0.013 (0.008–0.021)	
(95 % CI)	λ_2_: 0.154 (0.020–1.186)			λ_2_: 0.012 (0.007–0.019)		

Seropositivity and seroconversion rates (SCR) are presented for *P. falciparum* and *P. vivax*. If the best model to fit the age-adjusted seropositivity data was one with a change point in transmission, the SCR before the change point is indicated as λ_1_, while the SCR after the change point is indicated with λ_2_

^a^For 17 individuals age data was missing, therefore total numbers in the ‘all ages’ group differ slightly from those presented in the separate age categories

**Fig. 2:**
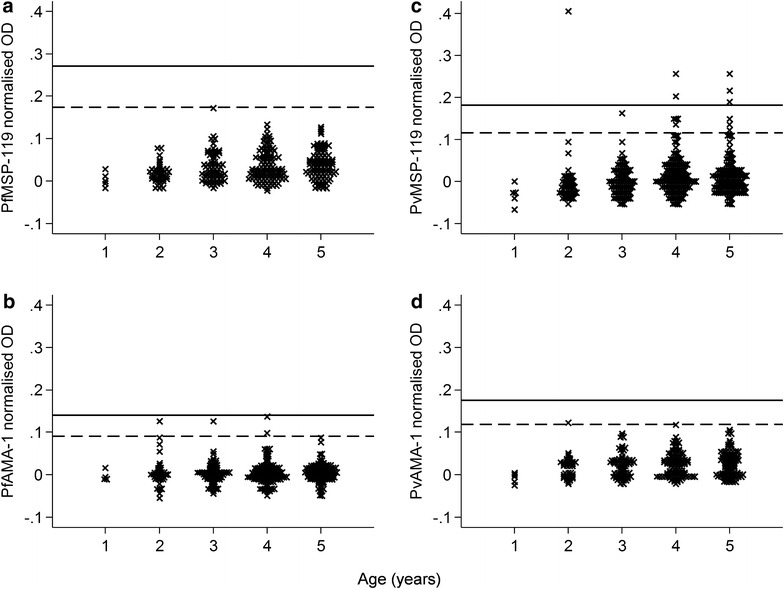
Distribution dot plot of antibody responses for children 1–5 years of age. Antibody levels are expressed as normalized OD values. *Horizontal lines* represent thresholds for seropositivity and are based on 3 (*dashed lines*) or 5 (*solid lines*) standard deviations (SD) plus the mean of the lower Gaussian distribution (see “Methods” section). Plots are divided by antigen: **a** PfMSP-1_19_, **b** PfAMA-1, **c** PvMSP-1_19_ and, **d** PvAMA-1. *OD* optical density, *Pv* *P. vivax*, *Pf* *P. falciparum*, *AMA*-*1* apical membrane antigen 1, *MSP*-*1*_*19*_ merozoite surface protein 1–19

In the 333samples tested from children aged one to 5 years only 18 were seropositive (*P. falciparum* 4/333, 1 % and *P. vivax* 15/333, 5 %). In the city, 70 % (7/10) and 40 % (4/10) were seropositive to PvMSP-1_19_ and PfAMA-1 antigens, respectively, while one of these samples showed seroreactivity to both antigens (Additional file 1). In the village, 100 % (8/8) and none was seropositive to *P. vivax* and *P. falciparum* antigens, respectively. Corrected OD values in these seropositive children never exceeded 0.410 and only two were positive for more than one of the tested antigens (2/18; 11 %). In addition, their corrected OD values were always in close proximity to the cut-off of the antigen that they tested positive for (Fig. 2). If a more conservative cut-off of five standard deviations plus the mean of the lower Gaussian distribution was used, only six out of the 18 children remained positive (33 %). The majority lived in villages (5/6) and all only remained positive for PvMSP-1_19_ (Fig. 2; Additional file 2).

Only adults (>15 years old) were seropositive to more than two antigens. The majority of participants were positive to a single antigen: 84 % in the city (95 positive to one antigen/113 positives to any antigen) and 76 % in the village (132/173). Regarding positivity to multiple antigens, only one individual in the city (1/113; 1 %) and three in the villages (3/173; 2 %) were positive to all four antigens. Similarly, for three antigens, this was six in the city (6/113; 5 %) and two in the villages (2/173; 1 %).

**Fig. 3:**
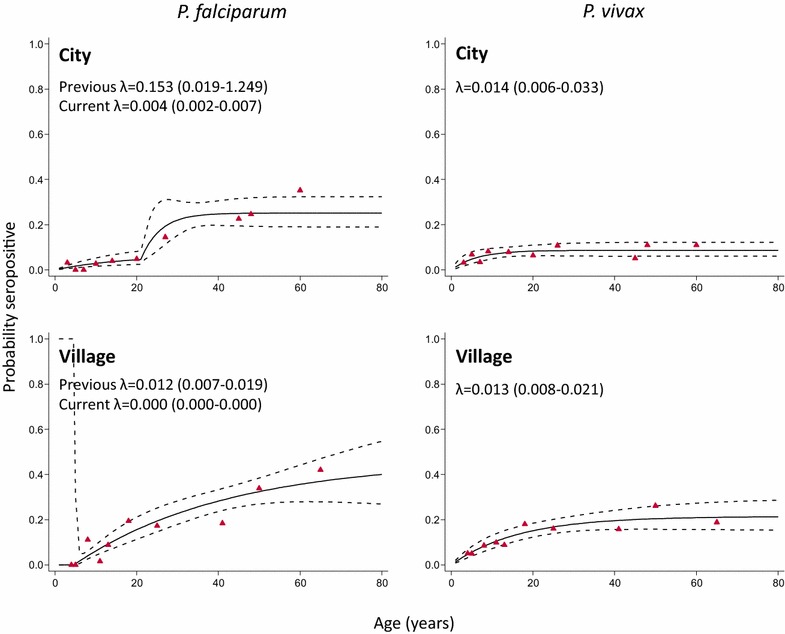
Anti-malarial seroconversion curves for participants in Chabahar city and surrounding villages, southeastern Iran. A reversible catalytic model was fitted to age-adjusted seropositivity data using maximum likelihood to create seroconversion curves. *Solid lines* represent the fitted probability for being seropositive, *dotted lines* represent the 95 % confidence interval for these fits, and triangles represent the observed proportion of seropositives per age decile. Seroconversion rates (SCR; λ) are presented on the graph. If the best model to fit the age-adjusted seropositivity data was one with a change point in transmission (see “Methods” section; Table 2), both SCRs are presented on the graph. The *P. falciparum* SRRs for the city and the village settings were 0.057 year^−1^ (0.034–0.094) and 0.010 year^−1^ (0.002–0.062), while for *P. vivax* these were 0.145 year^−1^ (0.049–0.423) and 0.048 year^−1^ (0.022–0.103)

Seroconversion curves are shown in Fig. 3. In the city a change point in *P. falciparum* transmission was seen at 21 years before sampling was done (*P* = 0.018) with a SCR of 0.004 year^−1^ (0.002–0.007) before the change point and 0.153 year^−1^ (0.019–1.249) after. Seropositivity to *P. falciparum* antigens among those under the age of 21 years was 2 % (10/411) and for those aged 21 or over 21 % (68/320). The SCR for *P. vivax* was 0.014 year^−1^ (0.006–0.033). In the villages, a change point in *P. falciparum* transmission was also seen, but more recently at 4 years before sampling (*P* = 0.039). The SCR before the change point was zero while after the change point it was 0.012 year^−1^(0.007–0.019). *Plasmodium falciparum* seroprevalence was none for those under the age of 4 years (0/46) and 16 % for those aged four or older (109/687). *Plasmodium vivax* transmission was similar to that in the city: 0.013 year^−1^ (0.008–0.021).

### Adjusted likelihood of seropositivity

Older age and lower SES were associated with *P. falciparum* seropositivity in the adjusted model, Table 3. The crude odd ratios (OR) of *P. falciparum* and *P. vivax* seropositivity for those living in a village compared to those living in the city were 1.49 (0.97–2.28, *P* = 0.068) and 2.06 (1.36–3.13, *P* = 0.001), respectively. The increased trend for *P. vivax* positivity remained apparent in the adjusted model (OR 2.12, 1.23–3.67, *P* = 0.009). In addition, for *P. vivax*, older age and reported history of malaria increased the likelihood of seropositivity significantly in the adjusted model. For *P. falciparum*, only age and SES were significantly associated with seropositivity in the adjusted model.

**Table 3:** Logistic regression analysis of explanatory factors for serological evidence of exposure to malaria

	*P. falciparum*					*P. vivax*				
	n/N	Crude OR (95 % CI)	*P* value	Adjusted OR (95 % CI)	*P* value	n/N	Crude OR (95 % CI)	*P* value	Adjusted OR (95 % CI)	*P* value
Location										
City	78/744	1	0.068	1	0.405	50/744	1	0.001	1	
Village	109/735	1.49 (0.97–2.28)		1.27 (0.71–2.26)		95/735	2.06 (1.36–3.13)		2.12 (1.23–3.67)	0.009
Age										
1 to 5	4/333	1	0.009	1	0.020	15/333	1	0.032	1	
6 to 15	21/404	4.51 (1.50–13.59)	<0.001	5.00 (1.31–19.16)	<0.001	33/404	1.89 (1.06–3.36)	<0.001	1.72 (0.88–3.39)	0.110
>15	161/725	23.48 (8.65–63.76)		22.17 (6.37–77.08)		96/725	3.24 (1.89–5.54)		2.55 (1.34–4.82)	0.006
LLIN use										
No	112/804	1	0.143	1	0.585	83/804	1	0.544	1	
Yes	73/669	0.76 (0.52–1.10)		1.13 (0.71–1.81)		62/669	0.89 (0.60–1.32)		1.07 (0.60–1.92)	0.820
Reported history of malaria										
No	129/1220	1	<0.001	1	0.156	101/1220	1	<0.001	1	
Yes	57/246	2.55 (1.62–4.00)		1.49 (0.85–2.60)		43/246	2.35 (1.71–3.22)		1.58 (1.10–2.28)	0.015
Breeding site^a^										
No	68/431	1	0.029	1	0.969	49/431	1	0.196	1	
Yes	119/1046	0.69 (0.49–0.96)		1.01 (0.49–2.10)		95/1046	0.78 (0.53–1.15)		1.10 (0.59–2.06)	0.753
Population density^b^										
Low	68/455	1	0.054	1	0.263	49/455	1	0.276	1	
High	83/763	0.69 (0.48–1.01)		0.83 (0.59–1.16)		65/763	0.77 (0.48–1.24)		0.97 (0.61–1.56)	0.901
Visitor from endemic setting										
No	161/1305	1	0.340	1	0.915	132/1305	1	0.269	1	
Yes	26/173	1.26 (0.78–2.03)		0.97 (0.50–1.86)		13/173	0.72 (0.40–1.30)		0.53 (0.24–1.18)	0.114
SES										
Low	80/506	1	0.122	1	0.011	47/506	1	0.969	1	
Middle	64/521	0.75 (0.51–1.09)	0.004	0.59 (0.40–0.88)	0.002	48/521	0.99 (0.63–1.57)	0.430	1.00 (0.58–1.74)	0.998
High	39/412	0.56 (0.38–0.81)		0.47 (0.31–0.73)		46/412	1.23 (0.73–2.07)		1.26 (0.72–2.20)	0.406

Confidence intervals were adjusted to allow for the clustered sampling

*OR* odds ratio, *CI* confidence interval, *LLIN* long-lasting insecticide treated net

^a^Within a 100 m radius of household

^b^High: more than ten houses within a 100 m radius of household; low: less than ten houses within a 100 m radius of household

## Discussion

Iran is certified as an elimination area since 2009 [11] and only approximately 1250 clinical cases were reported in 2014, of which the majority originated in the southeastern part of the country (CDMC, Tehran, Iran, unpublished data). As transmission reaches eliminating levels, identifying areas with remaining transmission or absent transmission requires new approaches. In this study, transmission levels of *P. falciparum* and *P. vivax* malaria were determined in Chabahar city and surrounding villages, Sistan and Baluchistan Province in the malaria elimination phase in southeastern Iran. There was no parasitological or serological evidence of recent local transmission, indicated by both an absence of microscopic or sub-microscopic (nPCR) infections, and weak or absent serological responses to either species in children up to the age of 5 years.

None of the children up to the age of 5 years in the villages showed seropositivity to *P. falciparum*, and only four were found in Chabahar city (2 %). Their adjusted antibody levels as represented by OD values were invariably low and close to the cut-off level, suggesting that they may not be related to recent infection or, given the age group, perhaps any infection. A number of alternative approaches are available to determine positivity thresholds in antibody tests [22] and indeterminate ranges are a common feature in commercially available serological assays [23]. When more conservative threshold of five standard deviations plus the mean of the lower Gaussian distribution was used none of the young children in the city was classified as seropositive. This suggests antibody responses in this age group were low and serological evidence of exposure to *P. falciparum* malaria is weak. Regarding *P.* vivax, using the more conservative threshold for positivity, only six out of the 15 seropositive 1–5 years old children remained positive. Similarly, for adults, serological responses were considered to be weak as only a minority mounted multi-antigenic responses. Approximately 15 % of the seropositives in the city were positive for more than one antigen and 25 % in the villages. Recent studies have likewise reported no parasitological evidence of transmission and low seroprevalence in the neighbouring malaria-endemic provinces of Hormozgan (Bashagard) and Kerman (Ghale-Ganj) [12, 14]. The detected lower overall seroprevalence in these studies is most likely due to lower levels of historical exposure. It is clear that for broader use of serological approaches in studies, regions and countries a standardized assay would be needed, such as those available in standardized anti-malarial antibody detection assays for the screening of blood products [24]). Nevertheless, the presented data from parasitological and serological tests strongly support low or absent levels of recent malaria transmission in these settings in Chabahar District.

The best model for age of *P. falciparum* seroconversion in Chabahar city was one with two forces of infection changing 21 years before sampling was done (*P* = 0.018). Seroprevalence in the city for individuals aged under the age of 21 was ten-fold lower compared to those aged 21 years and older. The timing of this change, 1991, coincides with a previously described dramatic drop in malaria cases in the early 90 s, both nationwide [25] and in the southwestern part of the country [13]. An alternative, but not mutually exclusive explanation, is that this could reflect increased risk of exposure in older individuals (>21 years), for example due to more frequent travel to malaria-endemic areas in neighbouring countries (e.g., Afghanistan and Pakistan). In the villages, a change point was seen at 4 years before sampling was done (2008; *P* = 0.039) suggesting transmission has only declined recently in these areas.

This might be explained by the up-scaling of different interventions during elimination strategies (CDMC, Tehran, Iran, unpublished data), such as active case detection, case management, early diagnosis, prompt and effective treatment [artemisinin combination therapy (ACT) plus single dose of primaquine on day 3 as the first-line recommended therapy for *P. falciparum* uncomplicated malaria and chloroquine with 8 weeks primaquine for radical treatment of *P. vivax*], indoor residual spraying, the distribution of LLINs, larviciding, and improved diagnostic capacities in health facilities (microscopy and rapid diagnostic testing) [26]. In addition, Iran is working to improve its cross-border collaborations and to apply a malaria early warning system and outbreak preparedness plan for epidemics by increasing training of microscopists, rural malaria mobile teams, and community volunteers to overcome this problem [27].

Moreover, no change in SCR was seen for *P. vivax*, with values similar in both rural and urban settings. This may be due to ongoing exposure to infection with this parasite or boosting of antibody levels by hypnozoite-derived infections. However, it may also be due to small sample size in addition to low seroprevalence [28]. This requires further investigation.

The overall *P. falciparum* and *P. vivax* seroprevalence presented here (13 and 10 %, respectively) was lower than previously described levels in other countries in the Eastern Mediterranean region, such as Somalia (18 and 19 %) [6], Djibouti (32 and 3 %, adults only) [29], and Yemen (32 and 3 %, children only) [30]. These studies took place between 2002 and 2011, while the data presented here are from 2012. The differences in seroprevalence are likely to be related to the lower level of transmission in Iran in recent years. Increasing collection of serological data in the region, alongside other metrics of malaria, has the potential to help in further characterizing regional transmission patterns.

As no parasite infections were detected with either microscopic or nPCR methods, risk factors to describe exposure to malaria could only be examined using serological outcomes. The increased likelihood of seropositivity to *P. vivax* remained twice as high for villagers in comparison with city dwellers in the adjusted model. It seems that ‘previous’ *P. vivax* transmission was higher in the villages surrounding Chabahar city than in the city itself, indicating higher potential receptivity for the re-introduction of malaria in the villages in this district. More geospatially explicit work would allow specific targeting of these areas to make their use more cost-efficient [31]. Iran has several intervention policies in place, such as the distribution of bed nets free of charge since 2005 [10], vector control strategies (such as microbial larviciding), as well as active case detection, case management, artemisinin-based combination therapy as the first-line recommended therapy for *P. falciparum* uncomplicated malaria, and improved diagnostic capacities. These could help maintain the absence or prevent the re-introduction of malaria transmission in the study areas with very active human population movements between Iran, Afghanistan and Pakistan, common parasite-vector fauna, as well as similar economic-cultural inhabitants, which provides an environment that is in favour of parasite transmission. Further monitoring of seroprevalence in residents of the study areas is considered highly useful given the low levels of transmission and population movement between malaria-endemic areas of Pakistan and Afghanistan that might affect transmission in these settings.

Interestingly, SES influenced the trend for seropositivity to *P. falciparum*, but not *P. vivax*. This observation might be due to *P. vivax* antibody levels being predominantly associated with relapses rather than new infections, as recently described in Papua New Guinea [32], and thus not influenced by household characteristics. The shown similarity in *P. vivax* (but not *P. falciparum*) seroconversion curves between city and village settings as well as the fact that there was weak to absent parasitological or serological evidence of recent *P. vivax* transmission in children, support this hypothesis. However, the latter may also be due to a higher risk of *P. vivax* in older individuals (e.g. due to work). It would be useful to confirm these findings in other *P. vivax* endemic, but eliminating, areas. These assumptions would be strengthened by inclusion of a broader range of antigens, ideally including those related with hypnozoite carriage (as yet unidentified).

## Conclusion

This study aimed to determine malaria transmission patterns in Chabahar District, southeastern Iran. As entomological endpoints are challenging to define correctly at very low transmission levels [4, 33], the study focussed on parasitological endpoints as well as serological measures, which are considered most appropriate in elimination settings [3, 9]. Firstly, the study found no evidence of recent transmission indicated by the fact that no parasite-carrying individuals were identified (by either microscopy or nPCR) and children showed weak or absent serological responses to either *P. falciparum* or *P. vivax*. Secondly, a change point in *P. falciparum* transmission in Chabahar city was detected that overlaps with a previously described dramatic drop in cases since the early 1990s [13, 25]. Lastly, the study showed a higher level of recent *P. vivax* transmission compared to *P. falciparum* transmission based on the SCRs in both the city and village settings. Whether these antibody responses reflect active transmission due to new infections or responses to relapse infections is unclear. The weak to absent evidence of recent malaria transmission in Chabahar District, even though this study targeted malaria prone areas, is promising in the context of elimination.

## Additional files

10.1186/s12936-016-1432-1 Characteristics of individuals that were categorized in socio-economic status (SES) tertiles based on principal component analyses.
10.1186/s12936-016-1432-1 Anti-malarial antibody responses for seropositive children 1 to 5 years of age in Chabahar city and surrounding villages.

## Abbreviations

SCR: seroconversion rates
CDMC: Center for Communicable Diseases Management and Control
LLIN: long-lasting insecticide-treated nets
nPCR: nested polymerase chain reaction
ssrRNA: small sub-unit ribosomal ribonucleic acid genes
PvAMA-1: apical membrane antigen-1 for *P. vivax*
PvMSP-1_19_: 19 kDa fragment of merozoite surface protein-1 for *P. vivax*
PfAMA-1: apical membrane antigen-1 for *P. falciparum*
PfMSP-1_19_: 19 kDa fragment of merozoite surface protein-1 for *P. falciparum*
SES: socio-economic status
OD: optical density
OR: odd ratio
