# Supplementary material for: Anti-malarial seroprevalence assessment during an elimination programme in Chabahar District, south-eastern Iran
Source: Malar J. 2016 Jul 22;15:382. doi: 10.1186/s12936-016-1432-1 (PMC4957887; doi:10.1186/s12936-016-1432-1)
Supplement: Supplementary file 1 — 10.1186/s12936-016-1432-1 Characteristics of individuals that were categorized in socio-economic status (SES) tertiles based on principal component analyses. [file 12936_2016_1432_MOESM1_ESM.docx]

**Additional file 1 Characteristics of individuals that were categorized in socio-economic status (SES) tertiles based on principal component analyses.**

1. **City**The first principal component accounted for 25% of total variation.

| SES | Individuals (no.) | Individuals (%) | Mean SES score | Percentage of individuals with: | | | | | | |
| --- | --- | --- | --- | --- | --- | --- | --- | --- | --- | --- |
|  |  |  |  | **Cement walls** | **Plaster ceiling** | **Domestic animal** | **Pipeline water** | **Electricity** | **Other facility: fan, TV or car** | **Mean family size** |
| Low | 265 | 36 | -6.04 | 30 | 30 | 3 | 0 | 85 | 79 | 6 |
| Middle | 261 | 36 | 0.11 | 94 | 6 | 6 | 0 | 100 | 100 | 6 |
| High | 203 | 28 | 0.45 | 94 | 0 | 73 | 4 | 100 | 100 | 5 |
|  |  |  |  |  |  |  |  |  |  |  |
| All | **729** | **100** |  | **71** | **13** | **24** | **1** | **95** | **92** | **6** |

1. **Villages**The first principal component accounted for 18% of total variation.

| SES | Individuals (no.) | Individuals (%) | Mean SES score | Percentage of individuals with: | | | | | | |
| --- | --- | --- | --- | --- | --- | --- | --- | --- | --- | --- |
|  |  |  |  | **Cement walls** | **Plaster ceiling** | **Domestic animal** | **Pipeline water** | **Electricity** | **Other facility: fan, TV or car** | **Mean family size** |
| Low | 241 | 34 | -3.90 | 10 | 0 | 64 | 56 | 100 | 94 | 5 |
| Middle | 260 | 37 | -0.48 | 86 | 0 | 70 | 65 | 100 | 100 | 6 |
| High | 209 | 29 | 0.70 | 100 | 7 | 96 | 100 | 100 | 100 | 7 |
|  |  |  |  |  |  |  |  |  |  |  |
| All | **710** | **100** |  | **63** | **2** | **76** | **72** | **100** | **98** | **6** |
