# Supplementary material for: Anti-malarial seroprevalence assessment during an elimination programme in Chabahar District, south-eastern Iran
Source: Malar J. 2016 Jul 22;15:382. doi: 10.1186/s12936-016-1432-1 (PMC4957887; doi:10.1186/s12936-016-1432-1)
Supplement: Supplementary file 2 — 10.1186/s12936-016-1432-1 Anti-malarial antibody responses for seropositive children 1 to 5 years of age in Chabahar city and surrounding villages. [file 12936_2016_1432_MOESM2_ESM.docx]

**Additional file 2. Anti-malarial antibody responses for seropositive children1 to 5 years of age in Chabahar city and surrounding villages.**OD values from ELISA readings are corrected for background receptivity by subtracting the average of blank wells per plate and normalized against a positive control curve per plate (see methods). OD values in bold represent those greater than the cut-off and therefore the individual is considered seropositive for the indicated antigen. Those that remained positive with a more conservative cut-off of 5 standard deviations plus the mean of the lower Gaussiandistribution are indicated with a star (*). OD: optical density; ELISA: enzyme-linked immunosorbent assay; Pv: *P. vivax*; Pf: *P. falciparum*; AMA-1: apical membrane antigen 1; MSP-1_19_: merozoite surface protein 1-19.

| **ID** | **Age** | **PfAMA-1**  (cut-off 0.091) | **PfMSP-1_19_**  (cut-off 0.174) | **PvAMA-1**  (cut-off 0.118) | **PvMSP-1_19_**  (cut-off 0.116) |
| --- | --- | --- | --- | --- | --- |
| *City* | | | | | |
| 1 | 2 | **0.125** | 0.058 | 0.031 | 0.000 |
| 2 | 3 | **0.125** | 0.101 | 0.025 | **0.168** |
| 3 | 4 | **0.098** | 0.053 | 0.073 | 0.023 |
| 4 | 4 | **0.138** | 0.019 | 0.022 | 0.000 |
| 5 | 4 | 0.004 | 0.130 | 0.006 | **0.142** |
| 6 | 4 | 0.019 | 0.041 | 0.018 | **0.150** |
| 7 | 4 | 0.023 | 0.049 | 0.000 | **0.149** |
| 8 | 5 | 0.010 | 0.018 | 0.000 | **0.146** |
| 9 | 5 | 0.025 | 0.040 | 0.021 | **0.261*** |
| 10 | 5 | 0.035 | 0.052 | 0.056 | **0.123** |
| *Village* | | | | | |
| 1 | 2 | 0.005 | 0.033 | **0.120** | **0.410*** |
| 2 | 4 | 0.045 | 0.074 | **0.118** | 0.108 |
| 3 | 4 | 0.034 | 0.043 | 0.045 | **0.258*** |
| 4 | 4 | 0.037 | 0.122 | 0.037 | **0.149** |
| 5 | 4 | 0.008 | 0.048 | 0.051 | **0.201*** |
| 6 | 5 | 0.017 | 0.085 | 0.054 | **0.185*** |
| 7 | 5 | 0.021 | 0.052 | 0.000 | **0.139** |
| 8 | 5 | 0.000 | 0.065 | 0.032 | **0.216*** |
